# Illness perceptions predict distress in patients with chronic kidney disease

**DOI:** 10.1186/s40359-021-00572-z

**Published:** 2021-05-07

**Authors:** Priscilla Muscat, John Weinman, Emanuel Farrugia, Roberta Callus, Joseph Chilcot

**Affiliations:** 1 Health Psychology Section, Psychology Department, Institute of Psychiatry, Psychology and Neuroscience, King’s College London, 5th floor Bermondsey Wing, Guy’s Campus, London, SE1 9RT UK; 2 Institute of Pharmaceutical Science, Pharmaceutical Sciences Clinical Academic Group, King’s College London, 5th floor, Franklin -Wilkins Building, 150 Stamford Street, London, SE19NH UK; 3 Nephrology Department, Mater Dei Hospital, Msida, Malta

**Keywords:** Chronic kidney disease, Illness perceptions, Treatment perceptions, Distress, Depression and anxiety

## Abstract

**Background:**

Patients diagnosed with chronic kidney disease (CKD) report increased distress associated with their clinical diagnosis. Distress in patients with predialysis CKD, has been linked to several adverse events; including increased risk of hospitalisation, early dialysis initiation and even death, suggesting that distress is a matter of great concern during routine care in predialysis CKD.

**Aims:**

The present study aimed to assess the nature of illness perceptions and the level of distress in a CKD cohort diagnosed with different stages of kidney disease. It also aimed to explore the correlates of distress and to create a model for distress and its associated predictors making use of hierarchical regression analysis.

**Methods:**

A sample of 200 patients diagnosed with Chronic Kidney Disease were recruited for this study from the nephrology outpatient clinics of Mater Dei Hospital, Malta. The participants were assessed for their; illness perceptions, treatment beliefs, level of depression and anxiety, coping style, as well as treatment adherence. Routine clinical information was also collected for participants, including a co-morbidity score.

**Results:**

A percentage of 33.5% of the participants reported moderate distress, whilst 9.5% reported severe distress. Stronger illness identity, a perception of timeline as being increasingly chronic or cyclical in nature, greater consequences and higher emotional representations were associated with more advanced stages of CKD. In contrast, lower personal and treatment control and poorer illness coherence were associated with more advanced stages of CKD. Results from the hierarchical regression analysis showed that illness perceptions contributed significantly to distress over and above the clinical kidney factors. Being female, having low haemoglobin and specific illness perceptions including; perceptions of greater symptomatology, longer timeline, low personal control and strong emotional representations, as well as resorting to maladaptive coping, were all significantly associated with distress symptoms. Nevertheless, illness perceptions accounted for the greatest variance in distress thus indicating that the contribution of illness perceptions is greater than that made by the other known covariates.

**Conclusion:**

Illness perceptions hold a principal role in explaining distress in CKD, relative to other traditional covariates. For this reason, illness perceptions should be addressed as a primary modifiable component in the development of distress in CKD.

## Background

Chronic kidney disease (CKD) is found to affect both the structure and the function of the kidney, resulting in the progressive and irreversible loss of kidney function, as the condition degenerates from early stages to later stages of CKD [1]. As the incidence and prevalence of kidney disease has doubled in the last ten years and is expected to continue rise in the future [2], the key to success lies in the realm of prevention and management of CKD in order to slow the progression, with the ultimate and desired goal of avoiding the development of End Stage Kidney Disease (ESKD) [3, 4].

A routinely used measure of kidney function is estimated glomerular filtration rate (eGFR) [5] which is defined as the volume of fluid filtered from the glomerular capillaries into the Bowman’s capsule per unit of time (mL/min/1.73m^2^). The eGFR is dependent upon several factors including age, sex and body size. Because of the central role of eGFR in the pathophysiology of complications, the disease is classified into five stages on the basis of eGFR: (stage 1) an eGFR more than 90 mL/min per 1·73 m^2^, (stage 2) an eGFR of 60–89 mL/min per 1·73 m^2^, (stage 3) an eGFR of 30–59 mL/min per 1·73 m^2^, (stage 4) eGFR of 15–29 mL/min per 1·73 m^2^ and (stage 5) an eGFR less than 15 mL/min per 1·73 m^2^.

As eGFR decreases and patients approach ESKD, they require more invasive treatment and resorting to lifestyle and dietary management is no longer sufficient to manage the symptoms associated with renal disease, dialysis is required. Dialysis allows small and middles sized-molecules to be removed from the blood (e.g. metabolic end-products) or through the peritoneum (e.g. removal of solutes), whilst also removing fluid, through the use of two basic modalities; haemodialysis (HD) and peritoneal dialysis (PD) [6, 7].

Individual cognitions, particularly personal illness representations have been found to influence the onset and prognosis of psychological distress in chronic illness [8]. Those illness beliefs are conceptualised within a framework of self-regulation known as the common-sense self-regulation model (CS-SRM) [9, 10]. According to the model, people make sense of a health threat by developing their own cognitive and emotional representations also known as illness perceptions (IP) or illness representations (IR) of a health threat [8, 11]. The illness representations are processed in three stages; the first stage, interpretation, takes place whereby the individual forms IR based on the demographics, knowledge or symptom perception, personal and familial experiences [12]. In stage two, coping, these IR enable the individual to make sense of the illness and to identify strategies to reduce the threat of illness or symptoms by guiding the coping and behavioural strategies so as to reduce the threat [11]. In the third stage, appraisal takes place whereby the individual then analyses the outcomes of the adopted coping strategies.

Research on illness perceptions in kidney disease has confirmed that patients' beliefs are associated with important outcomes, including depression, nonadherence, and even survival [13–15]. Proposed causal mechanisms to explain these poor outcomes in relation to depression include increased rates of inflammation, as well as non-adherence to therapy, an unhealthy lifestyle and poor nutrition [14, 16, 17]. In a study by Jansen et al. [18] which examined the variability of illness and treatment perceptions at different phases of CKD; patients on haemodialysis and peritoneal dialysis believed more strongly that their treatment controls their illness and perceived more illness and treatment consequences than predialysis patients.

Patients diagnosed with CKD report increased depression and anxiety associated with their clinical diagnosis [19]. Moreover, as the severity of CKD worsens, so does the level of depression [20–22] and anxiety [19, 23]. In a study by Knowles et al. [19] looking at the prevalence of distress in a CKD cohort; 17.5% of the patients had mild depression, whereas 7.5% had moderate depression severity, whilst 15% had mild anxiety, 8.8% had moderate anxiety, and the remaining 7.5% had severe anxiety, demonstrating that anxiety was the most common form of psychological distress. In addition, the findings of the study indicated that higher scores on illness perceptions (indicating poorer health perceptions) were associated with greater anxiety and depression symptoms and increased utilization of adaptive and maladaptive coping [19]. Studies exploring the role of coping amongst CKD participants suggest that maladaptive coping and defensive coping is associated with an increased anxiety and depression and lowered quality of life, whereas engagement of adaptive coping is associated with an attenuation of these symptoms [24].

In a recent study by Damery et al. [22] exploring the prevalence of patient distress in patients with end stage renal disease (ESRD), 33.3% reported mild to moderate distress, with 12.3% reported severe distress. The distress was found to impact negatively on quality of life and wellbeing. The prevalence of distress was significantly higher in younger patients, in women versus men and in black and minority ethnic patients versus patients of white ethnicity.

These studies provide evidence that poorer illness perceptions are associated with increased emotional concerns, lower quality of life, and reduced medication adherence. Whilst these studies have explored the impact of illness and treatment perceptions and distress in kidney disease, they focus mostly on end stage kidney disease thus leaving a dearth of literature. More literature is needed on earlier stages of CKD to confirm whether this association is also present in earlier stages and if so, psychological care needs to be integrated as part of the management of CKD and as part of the predialysis CKD routine care in order to potentially slow the progression of kidney disease and prevent deterioration to ESKD.

The present study aimed to assess the nature of illness perceptions and the prevalence of distress in a CKD cohort. It also aims to explore the correlates of distress looking at demographic and clinical kidney data, illness perceptions, treatment beliefs, coping and adherence with the aim of informing the final analysis. Finally, the study aimed to create a model for distress and its associated predictors making use of hierarchical regression analysis. Given past and related evidence from the literature, the following research questions were addressed:

- Do illness perceptions and treatment beliefs vary in patients diagnosed with different stages of Chronic Kidney Disease?
- What is the prevalence of distress in Chronic Kidney Disease?
- Which factors are associated with increased distress in a Chronic Kidney Disease cohort?
- What are the major predictors of distress in a Chronic Kidney Disease cohort?

In line with the research findings presented earlier, it was hypothesised that CKD patients with more advanced stages of the disease (Stage 4–5) would present with higher scores on negative illness perceptions (illness identity, chronic timeline, consequences and emotional representations) in comparison with those in early stages (Stage 1–3) and that the higher scores on those negative illness perceptions (indicating poorer health perceptions) would be associated with increased distress scores in chronic kidney disease. In addition, it was hypothesised that illness perceptions would play a crucial role in predicting distress.

## Methods

### Participants and procedure

A total of 250 patients diagnosed with Chronic Kidney Disease, were approached by the medical team to take part in this study via phone calls or during the Nephrology outpatient clinics at the Mater Dei Hospital, the main general hospital in Malta, an island with a population of 442,395 people [25]. The study cohort was formed of adult patients over 18 years of age diagnosed with Chronic Kidney Disease ranging from early stage to predialysis stage. In contrast, patients who had already commenced dialysis or have been referred for dialysis, transplanted patients, patients on conservative care and patients with evidence of severe cognitive impairment were excluded from this study. A standardized cognitive assessment was conducted using the mini mental state examination (MMSE) [26] and a score of < 23 was indicative of a level of cognitive impairment, in which case patients were excluded.

The 250 CKD patients were chosen randomly by the Consultant Nephrologists from the hospital data base of the Nephrology clinics. Of these, 220 provided informed consent to take part in the study of which 11 participants dropped from the study, 6 participants were ineligible to take part in the study as they had already commenced dialysis treatment and 3 were ineligible due to the presence of cognitive impairment as evidenced by the Mini Mental State Examination, MMSE.

### Disease assessment and questionnaires

The following demographic details were all recorded including; age, sex, ethnicity, time since diagnosis, CKD stage, treatment or nature of disease management, family status, and level of education. The following clinical data was included in the study; eGFR (calculated using the MDRD equation [27], Serum Creatinine (umol/l), Urea (mmol/l), Serum Albumin (g/l), Haemoglobin (g/dL), C Reactive Protein (CRP) (mg/L) and extra renal comorbidity score, assessed using the Charlson co-morbidity index, whilst adjusting for kidney disease by assigning one point for kidney disease to all participants [28].

The maltese version of the revised illness perception questionnaire (IPQ–R) [29], was used to assess illness representations of CKD. The IPQ–R has been validated for a range of chronic conditions including renal patients [30]. Illness identity was measured by the number of symptoms attributed to the kidney problem, summed up with a range of 0–14, with higher scores indicating increased illness identity. The following dimensions were measured on a 5-point Likert scale (strongly disagree to strongly agree): consequences (e.g. ‘my kidney problem has major consequences on my life’), emotional representations (e.g. ‘I get depressed when I think about my kidney problem’), timeline (e.g. ‘my kidney problem will last for a long time’), cyclical timeline (e.g. ‘my symptoms come and go in cycles’), illness coherence (e.g. ‘my kidney problem is a mystery to me’), personal control (e.g. ‘I have the power to influence my kidney problem’) and treatment control (e.g. ‘my treatment can control my kidney problem’). The possible range of scores for each dimension and the Cronbach’s alphas were presented in the results section. High scores on the timeline and consequence dimensions reflect strongly held beliefs about the chronicity and negative consequences of the condition, whilst the cyclical dimensions reflect strongly held beliefs that the condition is cyclical in nature. High scores on the personal control, treatment control and illness coherence subscales reflect strong perceptions of illness controllability and a greater personal understanding of the condition.

Beliefs about medicines questionnaire (BMQ) was used to assess beliefs about the treatment or medication [31]. Medication beliefs were evaluated using the BMQ which is divided into two sections: BMQ-General (sub-scales: Overuse and Harm) with 4 items scored on a 5-point likert scale and with scores ranging from 4 to 20 per subscale. BMQ-Specific (sub-scales: Necessity and Concerns), with 5 items per sub-scale with scores ranging from 5 to 25 per subscale. The answers were scored from 1 (strongly disagree) to 5 (strongly agree). Points of each scale were summed to give a scale score. During the study patients were asked about their beliefs with regards to phosphate binders or antihypertensive medication.

Medication adherence report scale 5 (MARS 5) [32, 33] was used to assess the level of adherence amongst participants assessed by 3 factors: medication adherence behavior, attitude towards medication, with reference to phosphate binders and/ or antihypertensive medication and general illness control. The scores range from 5 to 25.

Given the high correlation between patient health questionnaire 9 (PHQ-9) [34] and the generalized anxiety disorder 7-item scale (GAD-7) [35] a total distress score, termed the PHQ-ADS [36] was used for this study. The PHQ-ADS is the sum of the PHQ-9 and GAD-7 scores and can range from 0 to 48, with higher scores indicating higher scores of anxiety and depression symptomatology. The PHQ-ADS scale assesses different levels of distress. The following cut off scores were used; a score of 0–9 was used to outline minimal levels of distress, a score of 10–19 was used to outline mild levels of distress, a score of 20–29 was used to outline moderate distress, whilst a score of 30–48 outlined severe distress. This measure has been validated in a renal sample [16] showing that the PHQ-ADS is sufficiently unidimensional to warrant a total score.

Brief coping orientation to problems experienced questionnaire (Brief COPE) was used to assess the participant’s coping style [37]. The Brief COPE is the abbreviated version of the COPE Inventory and assesses dispositional as well as situational coping efforts. The 28-item Brief COPE consists of 14 subscales. The fourteen coping styles were clustered into two dominant coping styles as outlined in the model by Meyer [38] who grouped all eight problem-focused and emotion-focused coping categories under adaptive coping with scores ranging from 8 to 32 and all the six subscales of dysfunctional coping into maladaptive coping with scores ranging from 6 to 24. The Cronbach’s alpha scores were presented in the results section.

### Statistical analyses

Data analysis was carried out using SPSS v26. Statistical analyses were conducted on the data collected. The missing data was coded using 999 and included in the data analysis. Individual item missing data was very low (< 5%).

Descriptive analysis were undertaken so as to explore the demographic and the clinical characteristics of the sample.

Analysis of variance was used to explore the association between illness perceptions and disease stage, denoted by eGFR so as to determine whether there were differences in the nature of illness perceptions experienced across different stages of kidney disease.

Correlational analyses were undertaken to identify the correlates of distress in a CKD cohort, on exploring the association between demographic factors, clinical kidney factors, illness perceptions, treatment beliefs, coping and adherence, with distress scores. This analysis informed the construction of the distress model by outlining the significant associations in univariate analysis.

Hierarchical regression analysis was carried out to identify the predictors of distress amongst predialysis CKD patients. Multicollinearity was tested and the VIF values were well below 10 and the tolerance statistics were well above 0.2 [39] showing that there was no collinearity within the data.

## Results

### Demographic and disease characteristics

The mean age of the sample was 69.1 + 13.4 (range, 19–95). Table 1 outlines the demographic characteristics of the participants. Most of the participants recruited in the study were classified as stage 3 CKD with an eGFR range of 30–59 (48.5%, n = 97). Interestingly, the majority of the participants (37.8%, n = 71) have been diagnosed with CKD for the past two to five years, and only 21.2% (n = 40) were in their first year of diagnosis.

**Table 1:** Demographic characteristics of participants (N = 200)

Characteristic	Frequency	Percent (%)
*Sex*		
Female	75	37.5
Male	125	62.5
*Nationality*		
Maltese	192	96
Other	8	4
*Marital status*		
Single	12	6
Married or cohabitating	131	65.5
Separated or divorced	12	6
Widowed	45	22.5
*Level of education*		
Primary	93	46.5
Secondary	80	40
Tertiary	27	13.5
*Occupational status*		
Unemployed	4	2
Housewife	14	7
Retired	144	72
Employed	33	16.5
Student	3	1.5
*eGFR*		
60–90	16	8
30–59	97	48.5
15–29	53	26.5
10–14	34	17
*Time since diagnosis*		
Less than or equal to 6 months	20	10.6
More than 6 months or equal to 1 year	20	10.6
Around 2–5 years	71	37.8
Around 6–10 years	31	16.5
More than 10 years	46	24.5

Table 2 outlines the means and standard deviation for the clinical data of the participants within the sample including the; clinical kidney data, serum albumin, haemoglobin, CRP, extra renal comorbidity denoted by the Charlston Comorbidity score[28], and the levels of depression and anxiety in the sample, amounting to a total distress score. Finally the table also includes the treatment beliefs, the compliance scores and coping scores.

**Table 2:** Clinical data for the study sample

	N	Minimum	Maximum	Mean	SD
eGFR (ml/min/1.73m^2^)	200	10	86	33.64	16.86
Serum creatinine (umol/l)	199	56	812	227.70	146.61
Urea (mmol/l)	199	3	166	17.82	13.66
Serum albumin (g/l)	161	28	52	42.88	4.38
Haemoglobin (g/dL)	199	8	143	13.13	9.44
C-reactive protein (mg/L)	81	0	63	8.28	12.30
Charlston comorbidity score	200	0	17	4.89	3.10
Depression (PHQ9Score)	200	0	25	10.28	6.96
Anxiety (GAD7Score)	200	0	17	7.72	4.66
Distress (PHQ9ADSScore)	200	0	42	18.00	11.62
BMQ general	200	8	36	22.04	6.23
BMQ specific	196	20	41	30.21	4.14
MARS score	196	13	25	21.81	3.15
Adaptive coping	196	18	60	37.10	10.18
Maladaptive coping	196	14	41	26.07	7.24

### Illness perceptions (IP) and disease progression

Table 3 outlines the Cronbach’s alpha for all individual illness perception items and the mean scores and the standard deviations for the individual illness perception items across the whole sample and also presents the mean scores and standard deviations for participants diagnosed with early stage CKD classified with an eGFR of 30–90, in comparison with those of late stage, classified with an eGFR of 10–29.

**Table 3:** The association between CKD stage and IP items and item mean score + SD

Perception	Cronbach’s α	Possible range	Mean	SD	Stage 1–3 CKD normal to moderate eGFR (30–90 ml/min/1.73m^2^) (N = 113 /56.5%)		Stage 4–5 CKD low eGFR to ESRD (0–29 (ml/min/1.73m^2^) (N = 87/43.5%)		ANOVA
					Mean	SD	Mean	SD	*p* Value
1. Identity	N/A	0–14	3.74	3.3	2.23	2.1	4.51	3.2	*p* < 0.001
2. Timeline-acute/chron	0.95	6–30	22.25	6.2	20.10	6.5	24.02	5.2	*p* < 0.001
3. Timeline-cyclical	0.92	4–20	9.08	3.3	8.05	3.2	9.94	3.4	*p* < 0.001
4. Consequences	0.91	6–30	17.87	5.7	15.23	4.7	20.03	5.3	*p* < 0.001
5. Treatment control	0.82	5–25	16.05	3.9	17.27	3.4	15.49	3.2	*p* < 0.001
6. Personal control	0.84	6–30	18.55	4.6	19.97	4.1	17.94	3.6	*p* < 0.001
7. Illness coherence	0.79	5–25	16.98	3.7	17.30	4.1	16.80	2.6	*p* < 0.05
8. Emotion representations	0.94	6–30	19.90	6.3	17.36	5.8	21.80	5.6	*p* < 0.001

On running the analysis of variance on the individual illness perception subscales; the perceived identity, chronic timeline, cyclical nature, consequences and emotional representations were found to increase significantly from early stages (stage 1–3) to later stages of CKD (stage 4–5), whilst the personal control, the treatment control and the illness coherence were found to decrease from early stages to later stages of the disease as illustrated in Table 3.

Illness Perceptions did not significantly differ across the participant’s age groups, level of education and occupational status. This might be attributed to the fact that the majority of the participants were elderly patients (age; M = 69.1, SD = 13.4) who were mostly retired.

### Treatment beliefs and disease progression

On exploring whether treatment beliefs vary across the different stages of diagnosis from early stages (stage 1–3) to later stages of CKD (stage 4–5), the perceived necessity and perceived concerns were found to significantly increase (early stage; M = 29.45, SD = 3.9 to late stage; M = 31.19, SD = 4.2, *p* < 0.01) with decreasing kidney function as denoted by eGFR, thus suggesting that participants perceived their treatment as crucial in controlling their illness, but also reported increased concerns relating to the effects of the treatment as their kidney function declined. The other BMQ-General scales and the adherence score did not significantly differ from early stage to late stage CKD with *p* > 0.05.

### The frequency of distress

The frequency of distress was estimated using the PHQ-ADS score; 29% (n = 58) of participants reported minimal levels of distress, 28% (n = 56) of participants had mild levels of distress, 33.5% (n = 67) of the participants reported moderate levels of distress, whilst 9.5% (n = 19) reported severe distress with a 95% confidence interval (95% CI): 2.23–2.50.

### Correlates of distress

The following section will outline the univariate analysis linking the demographic, clinical kidney markers, illness perceptions, treatment beliefs and coping style with distress with the aim of outlining the correlates of distress.

### The relationship between demographic factors and distress

The correlations between demographic factors and distress were explored to determine which demographic factors were significantly correlated with distress. The following correlations were identified; age (r =  − 0.110, *p* > 0.05), sex (r = 0.156, *p* = 0.028), marital status (r = 0.098, *p* > 0.05), education (r =  − 0.068, *p* > 0.05), occupational status (r =  − 0.055, *p* > 0.05). Only sex was significantly correlated with distress. The other correlations failed to reach significance. This could be associated with the fact that most of the participants were elderly patients (age; M = 69.1, SD = 13.4) who were thus retired.

### The relationship between clinical kidney markers and distress

**Fig. 1:**
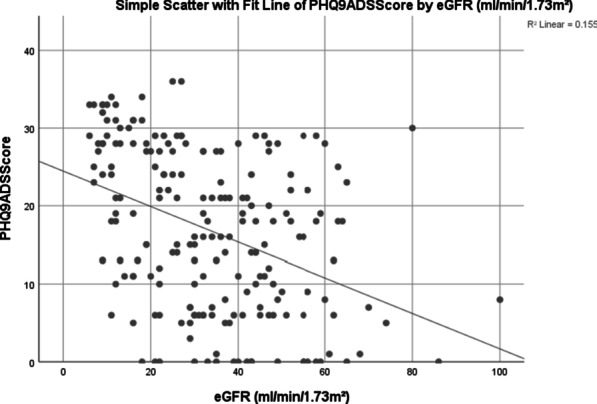
Scatterplot of distress versus kidney function

On correlating the clinical kidney data with distress, the level of distress was found to significantly increase as the condition progressed from early phase stage 1–3 CKD (M = 13.15, SD = 9.1), to late phase, stage 4–5 CKD (M = 21.52 SD = 9.8) with a *p* value < 0.001. Figure 1 outlines this association of distress versus kidney function as denoted by the eGFR. Table 4 presents the correlates of distress. The level of distress was found to correlate with eGFR with a magnitude of r =  − 0.374, *p* < 0.001.

**Table 4:** Correlates of distress

Clinical outcomes	Pearson correlation	Sig (2-tailed)
*Clinical markers*		
eGFR (ml/min/1.73m^2^)	− 0.374	0.000
Albumin (Serum)	− 0.173	0.030
Haemoglobin A1c	− 0.146	0.040
C-Reactive Protein mg/L	0.155	0.040
Charlston Comorbidity Score	0.111	0.119
*Illness Perceptions*		
IP-identity	0.481	0.000
Timeline acute-chronic	0.466	0.000
Consequences	0.618	0.000
Personal control	− 0.636	0.000
Treatment control	− 0.594	0.000
Illness coherence	− 0.272	0.000
Timeline cyclical	0.448	0.000
Emotional representations	0.619	0.000
*Treatment beliefs*		
BMQ general	0.236	0.001
BMQ general overuse	0.280	0.000
BMQ general harm	0.143	0.044
BMQ specific	0.324	0.000
BMQ specific necessity	0.359	0.000
BMQ specific concerns	0.211	0.003
*Brief COPE-2 factor model*		
Adaptive coping	− 0.678	0.000
Maladaptive coping	0.697	0.000
MARS5 score	− 0.143	0.044

As outlined in Table 4, eGFR, serum albumin, haemoglobin and CRP all correlated with distress. Interestingly enough the correlation for extrarenal comorbidity failed to reach significance.

### The relationship between illness perceptions and distress

**Fig. 2:**
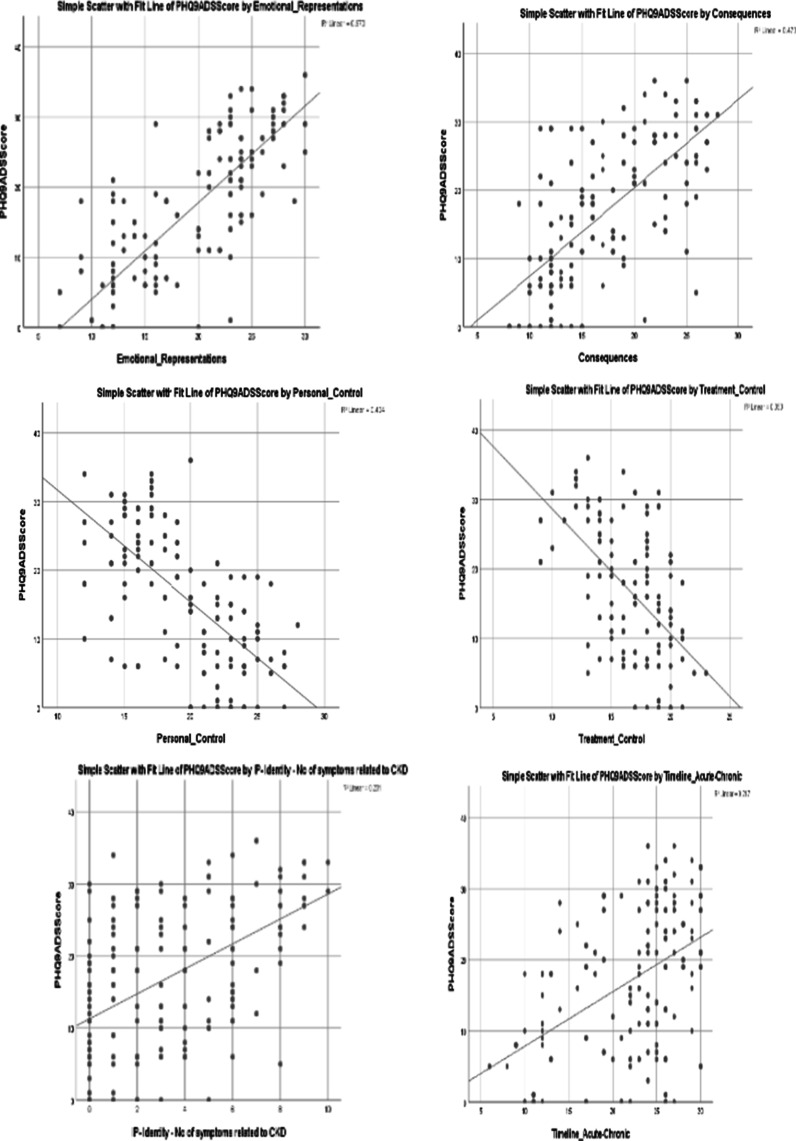
Scatterplot of distress versus illness perception items

Correlation analysis was also used to analyse the univariate associations between the individual illness perception items and distress. Table 4 indicates the Pearson Correlation and the level of significance for the association between illness perception items and distress. The strongest associations were that of distress with personal control (r =  − 0.636), emotional representations (r = 0.619), perceived consequences (r = 0.618), treatment control (r =  − 0.594), identity (r = 0.481) and timeline (r = 0.466). Figure 2 outlines the scatterplot of these associations.

As shown in Table 4, distress is positively associated with all the illness perception items, except for personal control (r =  − 0.636, *p* < 0.001), treatment control (r =  − 0.594, *p* < 0.001) and illness coherence (r =  − 0.272, *p* < 0.001) which were negatively related to distress, thus suggesting that the more personal control, treatment control and illness coherence, the lower the level of distress. In contrast, the more perceived illness and treatment consequences, and lower perceptions of control, the higher the associated distress.

### Treatment beliefs and distress

As outlined in Table 4, treatment beliefs were all positively correlated with distress. All correlations were significantly related, with the strongest correlations being; BMQ Specific (r = 0.324) and BMQ Specific Necessity (r = 0.359), and BMQ General (r = 0.236) and BMQ General Overuse (r = 0.280). Thus, suggesting that the more the participants felt that their health depended on the medications and the more they felt that doctors tend to overprescribe, the more they were distressed.

### Coping and distress

Correlation Analysis was also used to explore the association between distress and coping. Table 4 indicates that the use of adaptive coping was negatively associated with distress (r =  − 0.678, *p* < 0.001) whilst maladaptive coping was positively associated with distress (r = 0.697, *p* < 0.001). Thus, the more maladaptive the patient’s coping style, the higher the levels of distress amongst pre-dialysis CKD participants. The Cronbach’s alpha scores for this two-factor model were adaptive coping (α = 0.802) and maladaptive coping (α = 0.726). In addition, the level of adherence was negatively associated with distress (r =  − 0.143), thus suggesting that the more the participants were distressed, the less adherent they were.

### A model of distress for chronic kidney disease

#### The rationale

Following the correlation analysis which revealed the univariate associations of each of the factors with the dependent variable distress, hierarchical regression analysis was performed to investigate the predictive value of demographic and clinical kidney data, illness perceptions, treatment beliefs and coping and adherence on distress symptoms in CKD patients, with the aim of creating a model of distress.

#### The model structure

The following factors were included in the distress model:

##### Demographic and disease characteristics

With reference to the demographic characteristics that were included in the distress model; these were first analysed in univariate analysis namely; age, sex, marital status, education and occupational status. Of these, only sex was found to be significantly associated with distress. Upon loading the demographic factors, both sex and age were loaded in step 1. Age was included in the model even though it wasn’t significantly related to distress, since age has been identified as a potential covariate of distress and kidney decline in previous studies [5, 23]. It was thus included in the model to ensure that all the variance relating to age was accounted for in the model.

With reference to the disease characteristics that were included in the distress model; eGFR, serum albumin, haemoglobin and C-reactive protein (CRP) were all significantly related to distress in univariate analysis. However CRP (which is not as routinely measured) was not included in the final model due to the missing values in this marker with only 81 measures recorded from 200 measures. Thus eGFR, serum albumin and haemoglobin were loaded in step 1 together with age and sex.

##### Illness perception items

The Illness Perception items namely Identity, Timeline, Consequences, Personal Control, Treatment Control, Illness Coherence, Timeline Cyclical and Emotional Representations were all significantly associated with distress in univariate analysis and were thus loaded in step 2 of the distress model.

##### Treatment beliefs

The Treatment Beliefs including BMQ General Overuse, BMQ General Harm and BMQ Specific Necessity and BMQ Specific Concerns were all significantly associated with distress in univariate analysis and were thus loaded in step 3 of the distress model.

##### Coping and adherence

The Adaptive and Maladaptive Coping scales and the MARS5 Score were all significantly associated with distress in univariate analysis and were thus loaded in step 4 of the distress model.

This sequence was adopted in order to determine the contribution of illness perceptions, treatment beliefs, coping and adherence on accounting for traditional exploratory variables that is demographic and disease characteristics. Table 5 outlines the four steps in the Regression analysis. As outlined in Table 5, regression analysis was calculated for the following steps; Step 1—Demographic and Disease Characteristics (including age, sex, eGFR, Albumin, and Haemoglobin); Step 2—Illness Perception items (including Identity, Timeline, Consequences, Personal Control, Treatment Control, Illness Coherence, Timeline Cyclical and Emotional Representations); Step 3—Treatment Beliefs (including BMQ General Overuse, BMQ General Harm and BMQ Specific Necessity and BMQ Specific Concerns) and Step 4—Coping and Adherence (including Adaptive and Maladaptive Coping and the Adherence score).

**Table 5:** Hierarchical regression analysis for the predictors of distress

Variables	Step 1 (Model 1)			Step 2 (Model 2)			Step 3 (Model 3)			Step 4 (Model 4)		
	β	95% CI for β	Sig	β	95% CI for β	Sig	β	95% CI for β	Sig	β	95% CI for β	Sig
Age of Participants	− 0.15	− 0.26 to − 0.03	0.01	0.01	− 0.06 to 0.08	0.82	− 0.03	− 0.10 to 0.05	0.45	− 0.02	− 0.09 to 0.05	0.57
Sex	2.7	− 0.36 to 5.76	0.08	1.96	0.20 to 3.71	0.03	2.25	0.50 to 4.00	0.01	1.96	0.32 to 3.60	0.02
eGFR (ml/min/1.73m^2^)	− 0.2	− 0.28 to − 0.12	0.00	− 0.02	− 0.08 to 0.03	0.37	− 0.04	− 0.09 to 0.01	0.13	− 0.03	− 0.08 to 0.03	0.32
Albumin (Serum)	− 0.29	− 0.64 to 0.07	0.12	− 0.03	− 0.24 to 0.18	0.78	− 0.05	− 0.26 to 0.15	0.60	− 0.01	− 0.20 to 0.18	0.93
Haemoglobin A1c	− 0.41	− 0.52 to 1.35	0.38	− 0.38	− 0.16 to 0.92	0.16	− 0.47	− 0.07 to 1.01	0.09	− 0.52	0.02 to 1.02	0.04
IP Identity				0.69	0.34 to 1.05	0.00	0.66	0.30 to 1.01	0.00	0.58	0.24 to 0.92	0.00
Timeline Acute − Chronic				0.18	− 0.02 to 0.39	0.04	0.27	− 0.06 to 0.48	0.01	0.24	− 0.04 to 0.45	0.02
Consequences				0.07	− 0.21 to 0.35	0.62	0.12	− 0.16 to 0.40	0.41	0.12	− 0.14 to 0.38	0.35
Personal Control				− 0.51	− 0.79 to − 0.23	0.00	− 0.44	− 0.72 to − 0.16	0.00	− 0.32	− 0.58 to − 0.05	0.02
Treatment Control				− 0.12	− 0.55 to 0.31	0.58	− 0.12	− 0.55 to 0.31	0.59	− 0.06	− 0.51 to 0.39	0.79
Illness Coherence				− 0.01	− 0.30 to 0.28	0.95	− 0.04	− 0.34 to 0.27	0.81	0.08	− 0.22 to 0.39	0.59
Timeline Cyclical				0.23	− 0.06 to 0.51	0.12	0.02	− 0.31 to 0.34	0.92	0.08	− 0.25 to 0.40	0.65
Emotional Reps				1.01	0.77 to 1.25	0.00	1.00	0.77 to 1.24	0.00	0.84	0.61 to 1.07	0.00
BMQGeneral_Overuse							0.35	0.02 to 0.67	0.04	0.23	− 0.09 to 0.55	0.16
BMQGeneral_Harm							0.04	− 0.42 to 0.35	0.85	0.05	− 0.36 to 0.46	0.81
BMQSpecific_Necessity							0.40	− 0.08 to 0.88	0.10	0.42	− 0.02 to 0.86	0.06
BMQSpecific_Concerns							0.08	− 0.36 to 0.53	0.72	0.02	− 0.41 to 0.45	0.92
Adaptive Coping										− 0.09	− 0.24 to 0.06	0.25
Maladaptive Coping										0.29	0.08 to 0.50	0.01
MARS5 Score										0.14	− 0.16 to 0.44	0.36
		R^2^ = 0.199			R^2^ = 0.771			R^2^ = 0.790			R^2^ = 0.828	
		F(change) = 7.716			F(change) = 44.865			F(change) = 2.940			F(change) = 8.943	
		*p* < 0.001			*p* < 0.001			*p* < 0.05			*p* < 0.001	

### Findings

Whilst when loaded altogether the model accounted for 82.8% of the variance, the first model accounted for 19.9% of the variance in distress (R^2^ change = 0.199, F change = 7.716, *p* < 0.001). Participants who were younger (β =  − 0.15, *p* < 0.05) and who had lower eGFR (β =  − 0.2, *p* < 0.001) reported significantly higher levels of distress. Model 2 accounted for an additional 57.2% of the variance in distress (R^2^ change = 0.572, F change = 44.865 *p* < 0.001) after controlling for clinical factors and demographic characteristics, thus indicating that illness perception items were the strongest predictors of distress. Participants with the highest number of perceived symptoms (β = 0.69, *p* < 0.001), perceived timeline (β = 0.18, *p* < 0.05), low personal control (β =  − 0.51, *p* < 0.001) and stronger emotional representations (β = 1.01, *p* < 0.001) as their illness perceptions reported significantly higher levels of distress from their experience. Model 3 accounted for 1.9% of the variance (R^2^ change = 0.019, F change = 2.940 *p* < 0.05), indicating that the more perceived harm associated with the participant’s treatment regimen and the more the perceived necessity and concerns regarding their treatment, the higher the distress. Model 4, the coping style and adherence score accounted for 3.8% of the variance (R^2^ change = 0.038, F change = 8.943 *p* < 0.001), on controlling for the other factors. Therefore, participants presenting maladaptive coping strategies (β = 0.29, *p* ≤ 0.01) reported increasing distress.

The F statistic was found to increase significantly from model 1 to model 4, thus suggesting that all models were adding to the explained variance in distress. Illness perceptions were the most important predictors of depression and anxiety symptoms in patients with predialysis CKD. The final model showed that clinical kidney variables lost predictive value when illness perception variables were introduced.

## Discussion

The study aimed to assess the level of distress and to explore the nature and severity of illness and treatment perceptions experienced by patients diagnosed with CKD. 33.5% of the participants reported moderate distress, whilst 9.5% reported severe distress. This corroborates with the findings in dialysis patients by Damery et al. [23] who explored the prevalence of distress in patients with more advanced stages of kidney disease in end stage renal disease (ESRD) and reported a prevalence of 33.3% with mild to moderate distress, and 12.8% with severe distress. Thus the findings indicate that the levels of distress amongst patients with predialysis CKD are comparable to those in ESRD cohorts on dialysis.

Notwithstanding, the level of distress was found to increase on comparing early stages to later stages of CKD. This finding differs from the report of Hedayati et al. [40]. It could be speculated that these conflicting findings are due to differences in the measurement tools and classifications used. For instance, Hedayati et al. [40] used the Mini International Neuropsychiatric Interview Tool, which was distinct from our scale (PHQADS). Furthermore, the study by Hedayati et al. [40] classified CKD by four stages, which was also different from the classification system used in our study. Nevertheless, patients with more advanced stages of kidney disease typically experience a large number of symptoms [41]. The dread and uncertainties associated with potentially initiating dialysis treatment and the distress over one’s changing health status, may further aggravate depression and anxiety symptoms [42].

Despite the fact that depression and anxiety are increasingly prevalent among CKD patients, their detection and management are still not yet recognized in routine care of CKD [43]. This is resulting in increased risk of adverse events amongst patients with predialysis CKD; including early dialysis initiation, increased risk of hospitalisation, and even death [18, 40]. Therefore, it is crucial for healthcare providers to periodically evaluate the emotional status of CKD patients as they progress from early stages to more advanced stages to identify high risk cases earlier on.

On exploring the variability of illness and treatment perceptions across the different phases of CKD, stronger illness identity, a perception of timeline as being increasingly chronic or cyclical in nature, greater consequences and higher emotional representations were associated with more advanced stages of CKD. In contrast, lower personal and treatment control and poorer illness coherence were associated with more advanced stages of CKD.This could be due to the fact that the patients in earlier disease stages did not hold as strong beliefs about the illness as being as threatening and as evoking emotional response as those in the advanced stages. In contrast patients in later stages were aware of the fact that soon their current treatment will no longer suffice and perceived more consequences, longer timeline and more emotional representations.

The study also aimed to explore the correlates of distress and to create a model for distress and its associated predictors making use of hierarchical regression analysis. Illness perceptions were identified as the main predictors of distress amongst predialysis CKD. This result is supported by studies that show that illness perceptions contribute to depression and anxiety symptoms through changes in self-regulation and adjustment [13, 14, 40]. Illness perceptions that include pessimistic beliefs about illness identity or the severity of symptoms, a perception of a long and chronic timeline, low personal control and stronger emotional representations of disease were the most significant factors contributing to distress in predialysis CKD. This relationship confirms the theoretical and practical relevance of fostering positive and realistic illness perceptions when promoting emotional wellbeing in CKD. The association between illness perceptions and distress was highlighted by the significant correlations seen between all of the illness perceptions and distress. The level of distress correlated with all of the illness perception items and was positively associated with all the illness perception items, except for personal control, treatment control and illness coherence which were negatively related to distress, thus suggesting that the more perceived illness and treatment consequences, and lower personal control, treatment control and illness coherence, the higher the associated distress. This coincides with the findings of other studies in dialysis cohorts [19, 44–46].

In the final distress model, the significant predictors of distress were; sex thus revealing that females participants were significantly more distressed then males; low haemoglobin was also associated with distress, identity or the severity of symptoms, perception of a longer timeline, low personal control and stronger emotional representations. Maladaptive coping was also significantly associated with distress. Thus suggesting that the more maladaptive the patient’s coping style, the higher the level of distress Adaptive coping strategies tend to be associated with desirable outcomes and maladaptive coping strategies tend to be associated with undesirable outcomes including distress [38, 47]. The failure to observe any link between patients’ age and distress may also be partly due to the restricted age range of the participants and their older age (M = 69.1, SD = 13.4).

Much of the variance in distress was accounted for by illness perceptions and the final model revealed that on introducing illness perceptions in the model, clinical kidney variables (eGFR) lost their predictive value. Thus revealing that illness perceptions were more important than traditional explanatory variables in accounting for distress in kidney disease.

### Impact of the study

The present study shed more light on the prevalence of distress in predialysis CKD and yielded considerable evidence for the positive relationship between illness perceptions and distress. It has confirmed that the illness identity, the perceived chronic timeline, low personal control and strong emotional representations were significant predictors of distress in pre-dialysis CKD.

These findings provide additional support for the role of cognitive and emotional representations and the interplay with coping, in modulating depression and anxiety in CKD [9, 10]. In addition they support the theoretical relationship between a pattern of illness perceptions and mental health outcomes, Thus according to the results, by focussing on changing illness perceptions as a treatment goal, may improve psychological wellbeing and illness adaptation in CKD.

## Limitations

The study had a number of weaknesses, whilst being one of the first studies looking at illness perceptions and distress in early stage predialysis CKD patients; the sample was all recruited from the local nephrology outpatient clinics, thus capturing the service users that make use of government services only. In addition, being a cross sectional study, causality cannot be inferred. This study has also several strengths; a good sample of predialysis CKD participants and a good consent rate, a good response rate to questionnaires, since questionnaires were completed in the presence of the lead researcher and good matching of data, with clinical markers being collected in line with the psychological markers in terms of timeframe, so as to ensure congruence between the different factors.

## Conclusions

Therefore, it is possible to recommend that early diagnosis and treatment of depression and anxiety and close monitoring are especially important in CKD patients to improve their quality of life, whilst improving disease prognosis by delaying the start of dialysis.

In conclusion, illness perceptions hold a principal role in explaining distress symptoms in CKD, relative to other known covariates. For this reason, illness perceptions should be addressed as a primary modifiable component in the development of distress in CKD. Future primary and secondary prevention interventions should focus on the role of illness perceptions in the mental well-being of predialysis CKD patients.

In addition skilled attention to the patients’ illness perceptions and their levels of distress may enable health care providers to identify patients at risk for sub-optimal self-management and self-regulation, identify and focus on relevant problems as well as supporting healthy behaviour, self-management and self-care modalities of prevention which are crucial for improving the CKD prognosis.

## Data Availability

The data sets used and analysed during the current study are available from the corresponding author on reasonable request.

## Abbreviations

CKD: Chronic kidney disease
ESRD: End stage renal disease
CS-SRM: Common-sense self-regulation model
MMSE: Mini mental state examination
eGFR: Estimated glomerular filtration rate
CRP: C reactive protein
IPQ–R: Revised illness perception questionnaire
IP: Illness perceptions
BMQ: Beliefs about medicines questionnaire
MARS 5: Medication adherence report scale
PHQ-9: Patient health questionnaire
GAD-7: Generalized anxiety disorder 7-item scale
PHQADS: Patient health questionnaire and generalized anxiety disorder
Brief COPE: Brief coping orientation to problems experienced questionnaire
